# Insights from Amphioxus into the Evolution of Vertebrate Cartilage

**DOI:** 10.1371/journal.pone.0000787

**Published:** 2007-08-29

**Authors:** Daniel Meulemans, Marianne Bronner-Fraser

**Affiliations:** Division of Biology, California Institute of Technology, Pasadena, California, United States of America; Ecole Normale Supérieure de Lyon, France

## Abstract

Central to the story of vertebrate evolution is the origin of the vertebrate head, a problem difficult to approach using paleontology and comparative morphology due to a lack of unambiguous intermediate forms. Embryologically, much of the vertebrate head is derived from two ectodermal tissues, the neural crest and cranial placodes. Recent work in protochordates suggests the first chordates possessed migratory neural tube cells with some features of neural crest cells. However, it is unclear how and when these cells acquired the ability to form cellular cartilage, a cell type unique to vertebrates. It has been variously proposed that the neural crest acquired chondrogenic ability by recruiting proto-chondrogenic gene programs deployed in the neural tube, pharynx, and notochord. To test these hypotheses we examined the expression of 11 amphioxus orthologs of genes involved in neural crest chondrogenesis. Consistent with cellular cartilage as a vertebrate novelty, we find that no single amphioxus tissue co-expresses all or most of these genes. However, most are variously co-expressed in mesodermal derivatives. Our results suggest that neural crest-derived cartilage evolved by serial cooption of genes which functioned primitively in mesoderm.

## Introduction

The transition from sessile filter feeding to active predation in the vertebrate lineage was made possible by the evolution of a robust head skeleton. Embryologically, most vertebrate craniofacial cartilages and all pharyngeal cartilages are derived from the neural crest[1], a migratory and multipotent cell population formed at the edges of the nascent central nervous system. Data from invertebrate chordates suggest that the neural crest evolved from a population of migratory neural tube cells with limited developmental potential [2], [3]. Key to understanding the origins of the vertebrate head is understanding how these neural cells acquired the ability to form cellular cartilage.

**Figure 1:**
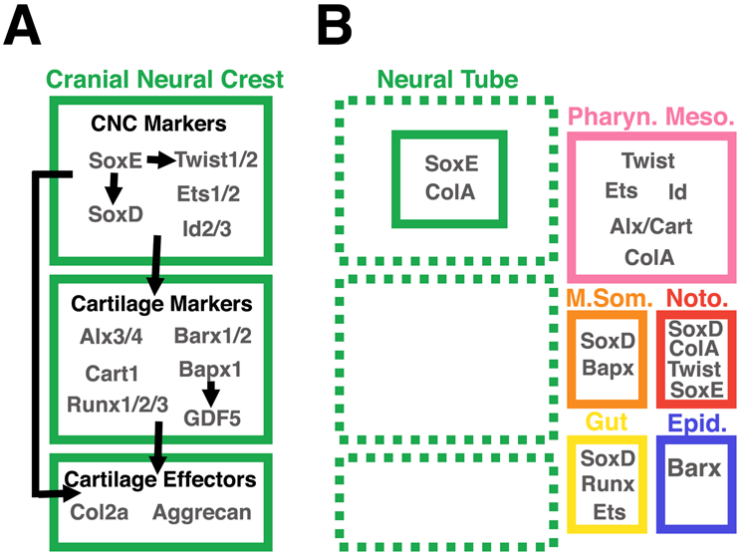
A provisional gene network operating in nascent neural crest-derived cartilage and expression of network component homologs in amphioxus. (A) We have classified genes in the network as cranial neural crest (CNC) markers, cartilage markers, or effector genes based on their expression, regulatory relationships, and biochemical functions. Among the CNC markers are *Sox9 (SoxE), Sox5/6 (SoxD), Twist1/2* and *Ets1/2* genes. All of these factors are expressed in post-migratory chondrogenic cranial neural crest [8], [9], [11], [45], [46]. *SoxE*, *SoxD*, and *Twist1/2* have been shown to cross-regulate, and to activate cartilage specifiers and effector genes. *SoxE* is required for expression of both *SoxD* and *Twist1/2* in migrating CNC [8], [34], while *Twist1/2* is necessary for the continued expression of *SoxE* in postmigratory CNC[9]. Both *SoxE* and *SoxD* cooperate to directly activate the definitive cartilage differentiation marker *Col2a1* in chondroblasts[47], while *Twist1/2* is required for expression of the *aristalless*-related transcription factors *Alx3/4* and *Cart1* [*9*]. *Ets1/2* expression overlaps temporally and spatially with *SoxE*, *SoxD* and *Twist1/2*, though functional relationships between it and the other network components have yet to be demonstrated [11]. In sea urchins, *Ets1/2* and *Alx3/4* orthologs are necessary for the formation of skeletogenic mesenchyme and are regulated by the same upstream factors, suggesting they cooperate in an evolutionarily ancient skeletogenic program [48], [49]. As chondrogenesis begins, presumptive pharyngeal chondrocytes express genes grouped here as cartilage markers (*Barx1/2, Alx3/4, Cart1, Runx1/2/3, Bapx1*, and *GDF5*). These genes are expressed in differentiating CNC-derived chondrocytes [12]–[14], [16], [21], [50]–[52], are downstream of CNC specfiers and upstream of effector genes like *Col2a1* and *Aggrecan, Barx1* physically interacts with *Sox9* to directly activate *Collagen2a1* expression [37]. As indicated above, *Alx3/4* and *Cart1* are regulated by *Twist1/2* [*9*]. *Runx1/2/3* expression in chondrocytes is dependent on *SoxE* function[16]. In the pharynx, *Bapx1* functions mainly to position the jaw joint by regulating expression of *GDF5* *[21]*, [53**]. In the mesoderm-derived axial skeleton, however, *Bapx1* is expressed broadly and operates upstream of *Sox9, Col2a1*, and *Runx1/2/3* [*54]*, [55**]. Essential for maintenance and establishment of the chondrogenic subnetwork are signaling molecules of the *FGF* and *Endothelin* families which are secreted by surround pharyngeal endoderm and ectoderm[20], [21] (not shown). These genes also mediate pharyngeal arch patterning by activating nested expression of various transcription factors in the nascent cartilages including *Dlx* and *Msx* genes, *Gsc*, and *Hand2* [21], [43](not shown). (B) The major expression domains of chondrogenic neural crest gene homologs in amphioxus neurulae and larvae. No single cell type expresses the complete set of vertebrate chondrogenic network genes, indicating the cranial neural crest cartilage program is a vertebrate novelty. Notably, most factors are expressed in mesodermal derivatives, suggesting neural crest-derived cartilage evolved via repeated cooption of primitively mesodermal genes.

Based on comparative morphology [4], [5] and the fossil *Haikouella* [*6*] it has been proposed that the first cartilages in the vertebrate head were pharyngeal cartilages of neural crest origin. In modern vertebrates, several genes mark post-migratory cranial neural crest cells as they populate the pharynx and form cartilage. These genes can be classified into three groups based on their expression patterns and demonstrated regulatory interactions (Figure 1A). The first set of genes is expressed broadly in neural crest cells during migration, and persists at high levels in post-migratory cranial neural crest. This group includes, but is not limited to, *Sox9* [*7*] (*SoxE*), *Sox5/6* [*8*] (*SoxD*), *Twist1/2* [*9*] *, Id2/3* [*10*], and *Ets1/2* [*11*] . All of these genes except *Ets1/2* have also been shown to be necessary for the formation of neural crest-derived cartilages. Expression of these factors precedes upregulation of several genes expressed in nascent chondrocytes and shown to be necessary for cartilage and bone differentiation. These genes include the transcription factors *Barx1/2* [*12*] *, Cart1* [*13*] *, Alx3/4* [*14*] *, Bapx1* [*15*], and *Runx1/2/3* [*16*] and the *TGF*-beta signaling molecule *GDF5* [*17*]. A third group of genes is expressed in differentiated cartilage and include classical markers of vertebrate cartilage like *Col2a1* [*18*] and the chondroitin sulfate-binding *lecticans* [*19*] *(i.e. aggrecan)*. Also essential for the differentiation of neural crest-derived cartilage are two classes of signaling molecules, *FGFs* [*20*] and *endothelins* [*21*]. These factors are secreted from adjacent pharyngeal endoderm and overlying ectoderm and are necessary for both cartilage differentiation and patterning via *Dlx, Msx*, *Hand2, Bapx1*, and *Gsc* transcription factors[21].

Gene expression studies suggest that the evolution of neural crest migratory ability and multipotency involved the cooption of transcription factors from other cell types [2], [10], [22], [23]. Genomic comparisons suggest this cooption also coincided with the evolution of new effector genes and signaling molecules[24] . While it is likely that the evolution neural crest-derived cartilage was driven by gene cooption it is unclear if this involved recruitment of individual genes into a novel gene regulatory network, or wholesale cooption of a pre-existing genetic program. Various chordate tissues have been proposed to represent evolutionary precursors of neural crest-derived cartilage, implying the utilization of pre-existing proto-chondrogenic gene networks. Cephalochordates possess skeletal elements reminiscent of vertebrate neural crest-derived pharyngeal cartilages. Though acellular, amphioxus pharyngeal gill bars are composed of fibrillar collagen[25], [26] and chondroitin sulfate[27]. They are also positioned between the endoderm and ectoderm and function to support the pharynx and express the cranial neural crest marker *Id* [*10*]. Based on these similarities, it has been suggested that the genetic network operating in cranial neural crest was recruited from collagen-secreting pharyngeal mesoderm[10] and/or endoderm[26], [28]. Like amphioxus gill bars, the notochords of urochordates, cephalochordates, and vertebrates also express fibrillar collagen[29], [30]. This has lead to speculation that a gene network mediating filbrillar collagen expression in vertebrate head cartilage was coopted from the notochord[30]. It has also been proposed that vertebrate neural crest-derived chondrocytes evolved from central nervous system (CNS) cells with the ability to express fibrillar collagen[31].

We tested whether vertebrate-like proto-chondrogenic gene programs could be operating in amphioxus tissues with proposed evolutionary relationships to the neural crest, including pharyngeal mesoderm, pharyngeal endoderm, neural tube and notochord. To this end we isolated amphioxus orthologs of 11 vertebrate genes involved in neural crest chondrogenesis and analyzed their expression patterns in embryos and larvae (summarized in Figure 1B). We find that no amphioxus cell type co-expresses orthologs of all or most vertebrate chondrogenic network components, arguing against wholesale cooption of a proto-chondrogenic gene program from any single cell type. Instead, our data suggests piecemeal assembly of the vertebrate cartilage gene network via repeated cooption of genes which functioned primitively in the mesoderm of the pre-vertebrate chordate.

## Results

### Identification of amphioxus neural crest and cartilage gene homologs

Using vertebrate protein sequences we BLAST searched an amphioxus EST database (Jr Kai Yu, unpublished results) for putative amphioxus orthologs of vertebrate cranial neural crest and cartilage genes. We identified amphioxus clones corresponding to vertebrate *Twist1/2, Ets1/2, Alx3/Alx4/Cart1, Runx1/2/3, Bapx1, FGF8/17/18* genes and a single amphioxus class A fibrillar collagen (*ColA*) (Table 1). Amphioxus genome release v1.0 (Joint Genome Institute) was searched for homologs of vertebrate *Sox5/6 (SoxD), Barx1/2*, and *GDF5/6/7*. Fragments of these genes were isolated by PCR. A single amphioxus *SoxE* cDNA was isolated by degenerate PCR and phage library screening. The amphioxus genome and EST database were searched exhaustively for potential amphioxus-specific duplicates of these genes, but none were found, indicating they are all present as single copies in the amphioxus genome. Putative orthology of each gene was suggested by BLAST searches of GenBank with amphioxus sequences and gene models. In each case, the highest identity hits were vertebrate or sea urchin homologs of the genes used to do the original searches. Orthology was further confirmed by phylogenetic analyses (Text S1, Figure S1, S2, S3, S4 and S5). Searches of the amphioxus genome with several vertebrate *endothelin* sequences yielded no similar sequences. Searches with vertebrate *aggrecan* sequences yielded two ESTs with high similarity to the c-terminal *EGF-lectin* modules of vertebrate *lecticans*.

**Table 1:** The genes analyzed in this study and their corresponding cDNA clones.

Vertebrate Proteins used for tBLASTn	Amphioxus Gene Models	EST clone names/clone origins
Zebrafish Aggrecan Rat Aggrecan	No clear ortholog	bfad033a24 CAXG15329
Chick Alx4 Mouse Cart1	Alx	bfne089p08
Chick Bapx1	Bapx	bflv046h23
Mouse Barx1	Barx	Exons amplified by PCR
Xenopus Col2a1	Col2a1 (ColA)	bflv014e16
Zebrafish Endothelin Chick Endothelin	None	None
Chick Ets2	Ets	bfad008i08
Chick FGF8	FGF8/17/18	CAXF12855
Chick GDF5	GDF5/6/7	Exons amplified by PCR
Chick Runx2	Runx1/2	bfne142d14
Chick Sox5	SoxD	cDNA amplified by PCR
N/A	SoxE	cDNA isolated by library screen
Xenopus Twist	Twist	bfne115j15

### Expression of the cranial neural crest marker orthologs, *SoxE, SoxD, Twist*, and *Ets*

**Figure 2:**
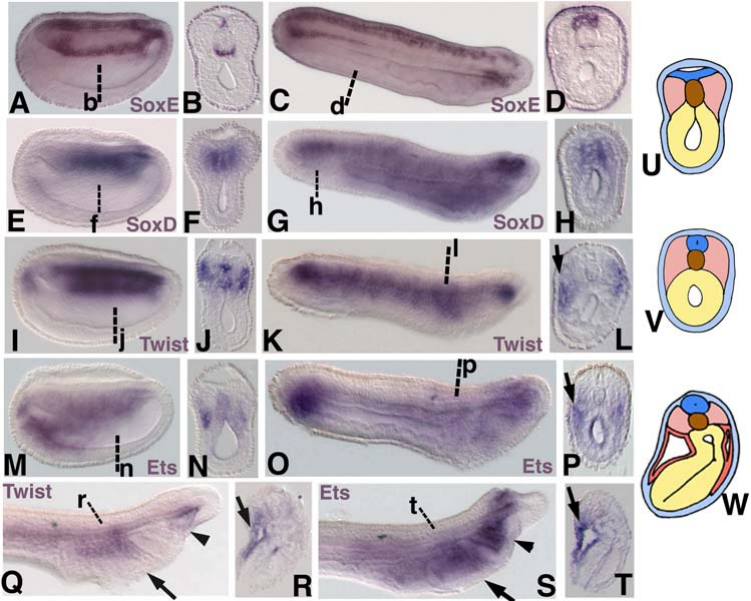
Expression of amphioxus *SoxE, SoxD, Twist*, and *Ets* at late neurula (15 h) and larval stages. In all panels showing wholemount specimens, anterior is to the left. (A) *SoxE* expression in ventral notochord and medial neural plate in late neurula. (B) Section through b in A. Superficial ectoderm staining is caused by adhesion of precipitate forming during the *in situ* hybiridzation procedure to the outside of the embryo. This artefact is distinguishable from actual signal because it is acellular, not visible in wholemount, and only readily apparent in overstained sections viewed using phase contrast optics. (C) *SoxE* expression throughout the neural tube and in ventral notochord cells at the anterior and posterior tips in early larva (24 h). (D) Section through d in C showing neural tube staining. (E) *SoxD* expression in the medial somites and notochord in late neurula. (F) Section through f in E. (G) *SoxD* expression in the posterior notochord, anterior gut, and cerebral vesicle of early larva. (H) Section through h in G showing notochord expression. (I)*Twist* expression in lateral somites and notochord in late neurula (J) Section through j in I. (K) *Twist* expression in ventrolateral mesoderm of early larva. (L) Section through the pharynx at l in K showing expression in pharyngeal mesoderm (arrow). (M) *Ets* expression in the posterior gut, anterior notochord, and ventral aspect of the anterior somites of late neurula. (N) Section through n in M. (O) *Ets* expression in the gut and anterior mesendoderm of early larva. (P) Section through the pharynx at p in O showing expression in pharyngeal mesoderm (arrow) and gut. (Q) *Twist* expression in the mesoderm of the first pharyngeal arch (arrow) and right gut diverticulum (arrowhead) of 1.5d larva. (R) Section through the first pharyngeal arch at r in Q showing mesodermal expression (arrow). (S) *Ets* expression in the mesoderm of the first pharyngeal arch (arrow), gut diverticulae (arrowhead), and cerebral vesicle of 1.5d larva. (T) Section through the first pharyngeal arch at t in S showing mesodermal expression (arrow). (U) Diagram of cross section midway through late neurula. (V) Diagram of cross section midway through early larva. (W) Diagram of cross section through first pharyngeal arch in 1.5d larva. In cephalochordate larvae, gill slits on opposite sides of the pharynx form asynchronously, with the right gill slits forming first. Thus, cross sections through the pharynx of amphioxus larvae reveal single gill bars rather than the symmetrical pharyngeal arches typical of analogous sections through vertebrate embryos. In U,V, and W, light blue is epidermal ectoderm, dark blue is the neural tube, brown is the notochord, yellow is endoderm, pink is somitic mesoderm, and red is pharyngeal arch mesoderm.

At neurula stages, amphioxus *SoxE* was observed in cells of the ventral notochord and medial neural plate (Figure 2A,B). In early larvae (24 h), *SoxE* expanded throughout the neural tube, but was lost from the ventral notochord (Figure 2D,C). *SoxE* expression was not detectable in late larvae. *SoxD* expression was seen in the nascent notochord and medial somite of neurulae (Figure 2E,F), then in the notochord, anterior gut, and cerebral vesicle of early larvae (Figure 2G, H). *Twist* expression was seen in the lateral somites and notochord of neurulae (Figure 2I,J), similar to *Twist* expression in the Chinese lancelet[32]. In early larvae, *Twist* transcripts were detected in the ventrolateral somites as they expanded to line the coelomic wall (Figure 2K,L). In late larvae, *Twist* expression was observed in the mesoderm of the first forming pharyngeal arch and right gut diverticulum (Figure 2Q,R). *Ets* expression was observed in the posterior gut and in the ventral part of the anterior somites at neurula stages (Figure 2M,N). At early larval stages expression was seen in the posterior gut, ventrolateral mesoderm, and the anterior gut diverticulae (Figure 2O,P). In late larvae, *Ets* was observed in the pharyngeal mesoderm of the first pharyngeal arch and the anterior gut diverticulae (Figure 2S,T).

### Expression of amphioxus orthologs of the chondrocyte markers *Alx3/Alx4/Cart1, Runx, Barx, Bapx*, and *GDF5*

**Figure 3:**
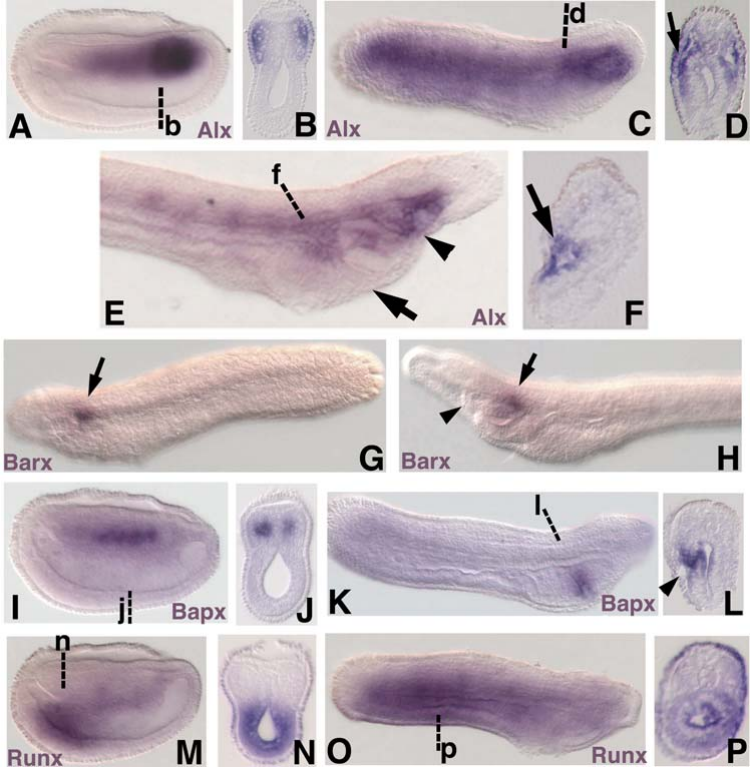
Expression of amphioxus *Alx, Barx, Bapx*, and *Runx* in late neurulae (15 h) and larvae. In all panels showing wholemount specimens, anterior is to the left unless otherwise indicated. (A) *Alx* expression in the lateral somites and gut diverticulae of late neurula. Strongest expression is seen in the gut diverticulae and first somites. (B) Section through the first somites at b in A. (C) *Alx* expression in ventral mesoderm and the anterior gut diverticulae of early larva (24 h). (D) Section through the pharynx at d in C showing expression in the pharyngeal mesoderm (arrow). (E) *Alx* expression in the mesoderm of the first pharyngeal arch (arrow) and the right gut diverticulum (arrowhead) of 1.5d larva. (F) Section through the first pharyngeal arch at f in E showing expression in mesoderm. (G) Anterior is to the left. Left side of an early larva focused in the plane of the ectoderm showing *Barx* expression in a patch of ectoderm (arrow) just caudal to the forming preoral pit. (H) Anterior is to the left. Left side of a 1.5d larva focused in the plane of the ectoderm showing *Barx* expression in a few ectodermal cells (arrow) caudal to the preoral pit (arrowhead). (I) *Bapx* expression in the medial somites of late neurula. (J) Section through j in I. (K) *Bapx* expression in a stripe of pharyngeal endoderm on the right side of an early larva approximating the region of the nascent first gill slit. (L) Section through l in K showing endodermal staining (arrowhead). (M) *Runx* expression in the posterior gut of late neurula. (N) Section through n in M. (O) *Runx* expression in the gut of early larva. (P) Section through p in O.

Amphioxus *Alx*, the ortholog of vertebrate *Alx3,Alx4*, and *Cart1*, was expressed in the lateral somites and strongly in the right gut diverticulum at neural stages (Figure 3A,B). In early larvae, *Alx* expression persisted in ventral somitic mesoderm and the right gut diverticulum (Figure 3C,D). In late larvae, expression of *Alx* was seen in pharyngeal arch mesoderm and the right gut diverticulum (Figure 3E,F). *Barx* expression was limited to a few ectodermal cells immediately caudal to the preoral pit at larval stages (Figure 3G, H). Amphioxus *Bapx* was expressed in the medial somite at embryonic stages (Figure 3I,J). In early larvae amphioxus *Bapx* marked a stripe of endoderm on the right side of the pharynx approximating the future location of the first gill slit (Figure 3K,L). Amphioxus *Runx* expression was seen in the posterior gut of neurulae (Figure 3M,N) and early larvae (Figure 3O,P) and diffusely in the late larval ectoderm (not shown). No detectable expression of amphioxus *GDF5/6/7* was observed in embryos or larvae up to day 4.5.

### Expression of amphioxus fibrillar collagen and *aggrecan*-like genes

**Figure 4:**
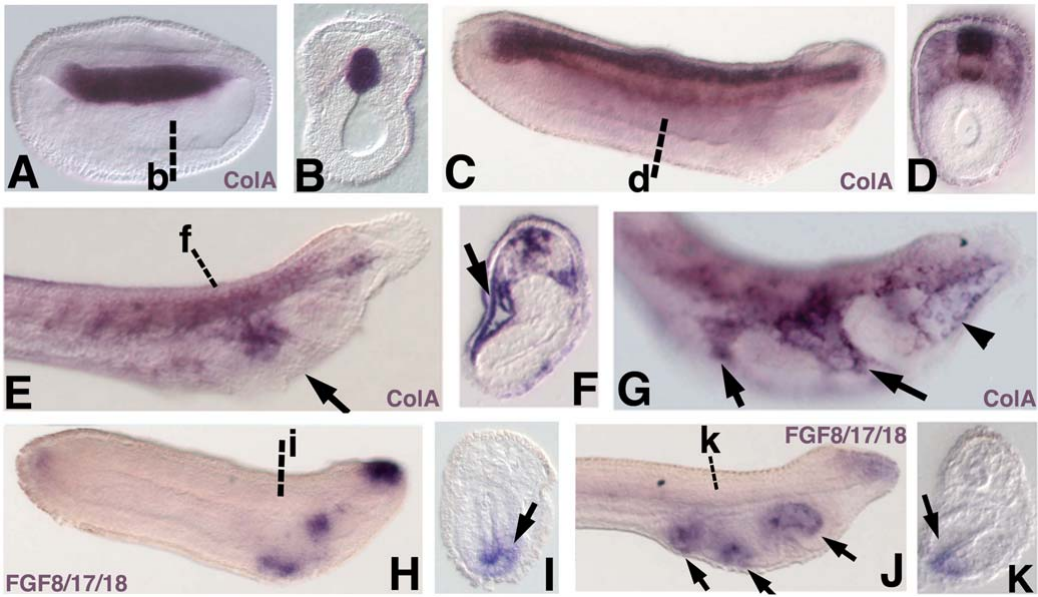
Expression of amphioxus *ColA*, and *FGF8/17/18* in late neurulae (15 h) and larvae. In all panels showing wholemount specimens, anterior is to the left. (A) *ColA* expression in the nascent notochord in late neurula. (B) Section at the level of b in A. (C) ColA expression in the neural tube and notochord in early larva (24 h). (D) Section through d in C showing weak expression in somitic mesoderm. (E) *ColA* expression in the mesoderm of the first pharyngeal arch (arrow) of 1.5d larva. (F) Section through the first pharyngeal arch at f in E showing mesodermal expression (arrow). (G) *ColA* expression in 2 gill slit larva. Strong expression is seen in the mesoderm of the first and second pharyngeal arches (arrows) and in individual cells of the right gut diverticulum (arrowhead). (H) *FGF8/17/18* expression in dorsal anterior ectoderm and two patches of pharyngeal endoderm. (I) Section through the pharynx at i in H showing *FGF8/17/18* expression in ventral endoderm (arrow). (J) *FGF8/17/18* expression in the pharyngeal endoderm of 1.5d larva (arrows). (K) Section through k in J showing expression in ventral endoderm (arrow).

Expression of the amphioxus ortholog of the definitive vertebrate cartilage marker *Col2a1* (Amphioxus *ColA*) in the embryonic (Figure 4A,B) and larval (Figure 4C,D) notochord and neural tube was similar to previous reports. However, we noted additional expression domains not previously described. Importantly, we saw strong expression of amphioxus *ColA* in the pharyngeal arch mesoderm of late larvae (Figure 4E–G), consistent with fibrillar collagen expression in the adult pharyngeal skeleton. We also observed weak embryonic expression of amphioxus *ColA* in the paraxial mesoderm of early larvae (Figure 4D). We did not observe expression of the *aggrecan*-like *c-lectin* domain clones *Agc-like1* or *Agc-like2* in amphioxus embryos or larvae.

### Expression of amphioxus *FGF8/17/18*

We also identified an ortholog of vertebrate *FGF8*, a signaling molecule secreted from pharyngeal endoderm required for pharyngeal chondrogenesis in vertebrates. At late neurula stages, amphioxus *FGF8/17/18* is expressed only in the cerebral vesicle (data not shown). In early larvae, expression was observed in two patches of pharyngeal endoderm (Figure 4H,I). Similar expression was seen in late larvae, corresponding to points of contact between the pharyngeal endoderm and ectoderm around the first and second gill slits and club-shaped gland (Figure 4J,K).

## Discussion

While gene expression data in itself is not evidence of gene regulatory relationships, the co-expression of non-secreted factors is a prerequisite for direct regulatory interactions. Thus, expression data can be used to falsify hypothetical gene regulatory interactions, such as those implicit in homology arguments. In this study we examined whether any tissue in the basal chordate amphioxus could be considered an evolutionary precursor, or latent homolog[33], of neural crest-derived cartilage. We reasoned that such a tissue should broadly co-express most factors required for neural crest chondrogenesis, including upstream transcriptional regulators and downstream markers of overt cartilage differentiation. As a starting point we focused on four tissues with proposed evolutionary relationships to neural crest-derived cartilage; the neural tube, pharyngeal mesoderm, pharyngeal endoderm, and notochord.

### Expression of amphioxus cartilage marker orthologs in the neural tube

Based on the expression of fibrillar collagens in parts of the vertebrate CNS, it has been proposed that vertebrate cartilage evolved from CNS cells with the latent ability to express fibrillar collagen [31]. Implicit in this scenario is that CNS cells in the prevertebrate chordate co-expressed orthologs of fibrillar collagen and the transcription factors which regulate it in cartilage. To test this we looked for neural expression of fibrillar collagen and its putative upstream regulators in amphioxus. We observed co-expression of amphioxus *ColA* and *SoxE* throughout the neural tube of early larvae with the exception of the cerebral vesicle, which lacks *ColA* expression. *SoxE* is then down-regulated before the mouth forms in late larvae, while *ColA* persists at least until the 2 gill slit stage. No other cartilage marker orthologs are broadly co-expressed with these two factors, though *SoxD* and *Ets* both label the larval cerebral vesicle. In vertebrate embryos *SoxE* genes (*Sox8, Sox9* and *Sox10*) are required for initial specification of the neural crest[34]. Later in post-migratory cranial neural crest cells, *SoxE* genes regulate the chondrogenic program and cooperate with *SoxD* and *Barx* factors to directly activate *Col2a1* [35]–[37]. Both these early and late functions of *SoxE* genes appear conserved to the base of the vertebrate lineage [38], [39]. The lack of broad co-expression of amphioxus *SoxE* with *SoxD* and *Barx*, argues against the presence of a vertebrate-like proto-chondrogenic program in the CNS of the prevertebrate chordate ancestor. However, the tight temporal and spatial co-expression of *SoxE* and *ColA* is consistent with an ancient gene-regulatory relationship between these genes in neural tissue.

### Expression of amphioxus cartilage marker orthologs in the pharyngeal mesoderm and endoderm

Electron microscopy and immunohistochemical analyses have revealed that, like vertebrate pharyngeal cartilages, the amphioxus gill bar skeleton is composed of fibrillar collagen and chondroitin sulfate [25]–[27]. This has lead to speculation that a vertebrate-like skeletogenic gene program operated in the pharyngeal endoderm[26], [28] or mesoderm[10] of the pre-vertebrate chordate. To evaluate these hypotheses we tested for broad co-expression of cartilage marker orthologs in the pharynx of amphioxus.

In the pharyngeal mesoderm of larvae we observed co-expression of *Twist*, *Ets*, *Alx*, and a homolog of the differentiated cartilage marker *Col2a1*. In vertebrates, *Twist1/2* and *Ets1/2* genes are expressed at high levels in post-migratory pharyngeal neural crest during pharyngeal arch formation. *Twist 1/2* has been shown to be necessary for these cells to form cartilage [9], [11] and *Ets1* regulates expression of *integrins* in chondrocytes [40]. The *aristalless*-related cartilage markers *Alx3*, *Alx4*, and *Cart-1* are similarly expressed in post-migratory pharyngeal neural crest and are required for chondrogenesis[13], [41]. Previous studies have not reported pharyngeal *ColA* mRNA expression at larval stages which would account for the presence of collagen protein in the gill bars [29], [30], though ColA transcripts are expressed broadly in pharyngeal ectoderm, endoderm, and mesoderm of adults [28]. We detect strong expression of *ColA* in larval pharyngeal mesoderm (Figure 4), suggesting amphioxus gill bars are initially mesodermal in origin. Thus, amphioxus pharyngeal mesoderm coexpresses orthologs of three transcription factors which regulate chondrogenesis in vertebrates, in addition to *ColA* and the cranial neural crest marker *Id*. While broad coexpression of these factors is suggestive of a rudimentary vertebrate-like chondrogenic program, amphioxus pharyngeal mesoderm does not express *SoxE* or *SoxD*, two factors essential for the formation of all vertebrate cartilages. This tissue also does not deploy orthologs of the vertebrate cartilage markers *Runx1/2/3*, *GDF5*, *Barx1/2*, or *Bapx1*. Furthermore, *Twist1/2* and *Alx3/4* mark lateral plate mesoderm in vertebrates, indicating their function in amphioxus pharyngeal mesoderm is not necessarily skeletogenic. Thus, while our data suggests some genes involved in vertebrate chondrogenic genes are expressed together with fibrillar collagen in amphioxus pharyngeal mesoderm, it is unclear if they function in a vertebrate-like chondrogenic gene network.

In the pharyngeal endoderm, we observed partially overlapping expression of *SoxD*, *Bapx*, and the signaling molecule *FGF8/17/18*. *SoxD* was expressed throughout the pharyngeal endoderm while *Bapx* mRNA was detected in a restricted domain approximating the region of the forming mouth. *FGF8/17/18* was observed in patches of ventral endoderm near the forming gill slits. In vertebrates, *FGF3* and *FGF8* are expressed in pharyngeal endoderm where they function to induce cartilage. Conserved expression of amphioxus *FGF8/17/18* in pharyngeal endoderm may indicate a conserved function in inducing pharyngeal skeletogenesis or patterning. However, the lack of broad coexpression of cartilage marker orthologs, including *ColA*, in amphioxus pharyngeal endoderm indicates this tissue does not deploy a vertebrate-type skeletogenic gene program at larval stages.

### A proto-chondrogenic differentiation program does not operate in the amphioxus notochord

Based on gross structural and biochemical similarities, and the expression of fibrillar collagen, it has been proposed that vertebrate cellular cartilage evolved by redeployment of a gene program which operated primitively in the notochord [38]. To address this possibility we assayed for coexpression of amphioxus orthologs of vertebrate cartilage markers in the notochord.

We observed expression of four transcription factors and *ColA* in the axial mesoderm of amphioxus. As previously reported, amphioxus *ColA* marks axial mesoderm until larval stages, mimicking *Col2a1* expression in vertebrates[38]. This expression overlapped to a limited extent with expression of *SoxE* in ventral notochord cells. However, broad expression of amphioxus *ColA* throughout the axial mesoderm implies that amphioxus *ColA* expression in this tissue is not *SoxE*-dependent as it is in neural crest-derived cartilage. *Twist* and *SoxD* are also coexpressed with *ColA* in the axial mesoderm of neurulae. *Twist* marks strips of ventral and dorsal notochord cells and is downregulated before larval stages. *SoxD* is broadly coexpressed with *ColA* , but like *Twist* , it is downregulated in early larvae. Notochord expression of *ColA* also overlaps with weak expression of *Ets* in the anterior notochord at neurula stages. In vertebrates, *SoxD* genes are necessary for *Col2a1* expression in notochord-derived cells[42]. Coexpression of amphioxus *SoxD* and *ColA* in the notochord may reflect an evolutionarily conserved regulatory relationship in axial mesoderm. Taken together, we find little evidence that a gene network resembling the neural crest chondrogenic program operates in the amphioxus notochord. However, it is possible that both the amphioxus notochord and cranial neural crest cells utilize *SoxD* genes to regulate fibrillar collagen expression.

### *Lecticans* and *endothelins* are vertebrate novelties associated with the evolution of cartilage

In vertebrate cartilages, *lecticans* are the major chondroitin sulfate-binding proteins. We could not identify clear amphioxus orthologs of *lecticans* (i.e. *aggrecan*) in the amphioxus genome. We did isolate two EST clones similar to the c-terminal domain of vertebrate *lecticans* (Tab. 1), but neither was expressed in embryos or larvae. Histological and biochemical assays demonstrate that amphioxus gill bars contain acid mucopolysaccharides and chondroitin sulfate[27]. It is possible that genes structurally related to *lecticans*, but not strictly orthologous to them, bind chondroitin sulfate in amphioxus. In vertebrates, *endothelins* are secreted molecules which induce and pattern pharyngeal arch cartilages[43]. We did not find a clear homolog of vertebrate *endothelins* in the amphioxus genome. Like the *lecticans*, this class of genes may represent a vertebrate novelty associated with the evolution of cellular cartilage. Recent genomic comparisons confirm the absence of *endothelins* in protochordates and indicate that other families of signaling molecules associated with neural crest migration and differentiation are unique to vertebrates[24]. Thus, the evolution of chondrogenic neural crest is associated with the cooption of evolutionarily ancient transcriptional regulators, as well as the appearance of novel downstream effector genes and signaling molecules like *lecticans* and *endothelins.*

### *De novo* assembly of the vertebrate CNC cartilage program via cooption of primitively mesodermal genes

It is unknown how neural crest cells acquired the genetic machinery necessary to form cellular cartilage. One possibility is this occurred relatively rapidly by wholesale cooption of an ancient chondrogenic program. Alternately, chondrogenic ability could have evolved gradually in neural crest cells via piecemeal cooption of individual genetic components. We find that no embryonic or larval tissue in amphioxus co-expresses all or most cartilage network orthologs, supporting *de novo* assembly of the vertebrate chondrogenic neural crest gene program. Though it is possible that some cartilage network orthologs are re-deployed together after metamorphosis, we view this as unlikely since most amphioxus tissue types, including the primary gill bars, form during larval stages[44].

Assuming that the vertebrate chondrogenic gene network is unique to vertebrates, we asked what the primitive function of these genes may have been in the vertebrate ancestor. While expression patterns alone do little to inform the precise functions of genes, similar expression of orthologous genes across related phyla often reflects conserved functional relationships. As mentioned above, *SoxE* and *ColA* both mark the neural tube in amphioxus, suggesting a vertebrate-type regulatory relationship between these genes in neural tissue predates the evolution of vertebrate cartilage. In adult hemichordates, which lack a central nervous system, *SoxE* and *ColA* are also coexpressed in pharyngeal endoderm, indicating this regulatory cassette may have evolved before the origins of chordates.

In both sea urchins and vertebrates, *Alx* and *Ets* genes are expressed in skeletogenic mesenchyme. Expression of amphioxus *Alx* and *Ets* genes in pharyngeal mesoderm which gives rise to collagenous skeletal elements may reflect conservation of an ancient deuterostome skeletogenic gene program coopted by neural crest cells. Functional studies will reveal if these genes act to regulate gill bar formation in amphioxus and if they can be considered components of a primitive chordate skeletogenic gene program.

In addition to *Alx* and *Ets*, we found that most other amphioxus orthologs of vertebrate CNC and cartilage markers were expressed in mesodermal derivatives, while relatively few genes mark epidermal, neural, or endodermal cells (Figure 1B). Similar expression of vertebrate chondrogenic neural crest markers in mesodermal derivatives suggests that most components of the vertebrate neural crest cartilage program operated primitively in mesoderm. This is consistent with the overlapping developmental potentials of cranial neural crest cells and mesoderm to generate connective tissue, muscle, and cartilage. In sum, our data suggests that the vertebrate chondrogenic program likely evolved via serial cooption of primitively mesodermal genes to neural crest cells in the first vertebrates.

## Materials and Methods

Vertebrate protein sequences were used to BLAST search an amphioxus EST database (Jr Kai Yu, unpublished results) for putative amphioxus orthologs. Access to library clones was kindly provided by Drs.J.K. Yu and Linda Holland. Degenerate PCR using primers against vertebrate *Sox8*, *Sox9*, and *Sox10* (SoxE5′1: TACGAYTGGWCIYTNGTNCCIATGCC, SoxE3′1:GGCTGRTAYTTRTAITCIGGRTRRTC) followed by phage library screening (library a gift of Jim Langeland) was used to isolate an amphioxus *SoxE* ortholog. Amphioxus genome release v1.0 (Joint Genome Institute) was searched for putative orthologs of vertebrate *SoxD, Barx1*, and *GDF5*. Fragments of these genes were isolated by PCR using diluted phage library and a vector-specific primer (*SoxD*) or genomic DNA (*Barx, GDF*). Forward and reverse primer sequences were:

SoxD5′: CCCCACATCAAGCGGCCAATGAATG

BarxEx15′: TATAGCTGGTTGTGCCTCTTG

BarxEx13′: AACATTCTACACACTGCGACG

BarxEx2 5′: GGAGGAGTTTACAGAGAGTAAC

BarxEx2 3′: ACAAGTCTTGTTGTGACCTGTAC

BarxEx3 5′: GCAATTAGCCTACGGACAC

BarxEx3 3′: GCGTGTTCCGATTAGTACAG

GDF5Ex1 5′: GAAAGGGGTAGATTGATTTCTTTTC

GDF5Ex1 3′: TACAGCCTTGTCGACGAAC

GDF5Ex2 5′: ATTTTGAACAGCTGCCGGG

GDF5Ex2 3′: CTGGGGTTCATGGAGTTG

For each amphioxus gene, *in situ* hybridization was performed on embryos and larvae ranging from 12 hour early neurula to 2.5d feeding larva (2–3 gill slits) as described previously. In the cases of *Barx* and *GDF*, *in situ* probes made against their amplified exonic sequences were pooled and used together. Embryos were embedded in 20% gelatin in Phosphate Buffered Saline and cryostat sectioned. Wholemount embryos in 40% glycerol/PBS, and sections, were photographed using a Zeiss AxioSkop 2 Plus. Images were processed using Adobe Photoshop.

## Supporting Information

Text S1 (0.81 MB DOC)

Figure S1 (1.40 MB TIF)

Figure S2 (1.91 MB TIF)

Figure S3 (1.57 MB TIF)

Figure S4 (1.40 MB TIF)

Figure S5 (0.69 MB TIF)
