# Supplementary material for: Insights from Amphioxus into the Evolution of Vertebrate Cartilage
Source: PLoS One. 2007 Aug 29;2(8):e787. doi: 10.1371/journal.pone.0000787 (PMC1950077; doi:10.1371/journal.pone.0000787)
Supplement: Text S1 — (0.81 MB DOC) [file pone.0000787.s001.doc]

# Supplemental Methods

Translated cDNA sequences and amphioxus genome release v1.0 gene models were aligned to published deuterostome, cnidarian (*Nematostella vectensis*), and *Drosophila melanogaster* protein sequences using ClustalX [1]. Bootstrapped Neighbor-Joining trees [2] were then constructed using ClustalX, and drawn using NJplot [3]. Bootstrap values were calculated from 1000 resamplings of the alignment data. Maximum Likelihood trees and quartet-puzzling reliability scores were generated from the same alignments using TREE-PUZZLE [4]. For most trees, cnidarian or *Drosophila* sequences served as outgroups. For others, related non-homologous deuterostome proteins were chosen.

# Supplemental Figure Legends

**Figure S1.** Phylogenetic analyses of amphioxus *Alx* and *Bapx*. (A) Bootstrapped Neighbor-Joining tree of deuterostome and *Drosophila* *Alx* homologs. The related *Drosophila* *retinal homeobox* (Rx) gene serves as an outgroup. (B) Maximum Likelihood tree generated using the same alignment as in (A). (C) Bootstrapped Neighbor-Joining tree of vertebrate, amphioxus, *Drosophila* and cnidarian *Bapx/Bagpipe* homologs. The cnidarian *Bapx* homolog *NK3* serves as the outgroup. (D) Maximum Likelihood tree generated using the same alignment as in (C). In all analyses the amphioxus gene groups strongly with its vertebrate or invertebrate deuterostome homologs, supporting orthology. Bootstrap values and quartet-puzzling reliability scores are shown at the branch points, and distance units are shown in the upper right hand or lower left hand corners of the trees. Gene names are prefixed by the initials of their respective species names. Species included in the analyses are *Drosophila melanogaster, Nematostella vectensis, Strongylocentrotus purpuratus, Paracentrotus lividus, Branchiostoma floridae, Danio rerio, and Mus musculus*.

**Figure S2.** Phylogenetic analyses of amphioxus *Barx, BarH,* and *Ets*. (A) Bootstrapped Neighbor-Joining tree of deuterostome and *Drosophila* *Barx and BarH homologs*. The distantly related homeobox genes *Drosophila* *BSH* and zebrafish *Dlx2,* serve as outgroups. (B) Maximum Likelihood tree generated using the same alignment as in (A). (C) Bootstrapped Neighbor-Joining tree of deuterostome and *Drosophila* *Ets* homologs. (D) Maximum Likelihood tree generated using the same alignment as in (C). In all analyses the amphioxus gene groups strongly with its vertebrate or invertebrate deuterostome counterparts, supporting orthology. Bootstrap values and quartet-puzzling reliability scores are shown at the branch points, and distance units are shown in the upper right hand or lower left hand corners of the trees. Gene names are prefixed by the initials of their respective species names. Species included in the analyses are *Drosophila melanogaster, Nematostella vectensis, Saccoglossus kowalevskii, Oikopleura dioica, Ciona intestinalis, Strongylocentrotus purpuratus, Paracentrotus lividus, Branchiostoma floridae, Danio rerio, Xenopus laevis, Gallus gallus, and Mus musculus*.

**Figure S3.** Phylogenetic analyses of amphioxus *FGF8/17/18* and *GDF5/6/7*. (A) Bootstrapped Neighbor-Joining tree of chordate and cnidarian *FGF8/17/18* homologs. The distantly related zebrafish *FGF3* gene serves as an outgroup. (B) Maximum Likelihood tree generated using the same alignment as in (A). (C) Bootstrapped Neighbor-Joining tree of chordate and cnidarian *GDF5/6/7* homologs. The distantly related *TGFß* genes amphioxus *BMP2/4* and zebrafish *BMP4* serve as outgroups. (D) Maximum Likelihood tree generated using the same alignment as in (C). In all analyses the amphioxus gene groups with its vertebrate homologs, supporting orthology. Bootstrap values and quartet-puzzling reliability scores are shown at the branch points, and distance units are shown in the upper right hand or lower left hand corners of the trees. Gene names are prefixed by the initials of their respective species names. Species included in the analyses are *Drosophila melanogaster, Nematostella vectensis, Branchiostoma floridae, Ciona intestinalis, Danio rerio, Homo sapiens, Xenopus laevis, Gallus gallus, and Mus musculus*.

**Figure S4.** Phylogenetic analyses of amphioxus *SoxD* and *SoxE*. (A) Bootstrapped Neighbor-Joining tree of deuterostome and *Drosophila SoxD* homologs. The distantly related amphioxus *SoxB2* gene serves as an outgroup. (B) Maximum Likelihood tree generated using the same alignment as in (A). (C) Bootstrapped Neighbor-Joining tree of deuterostome and *Drosophila SoxE* homologs. (D) Maximum Likelihood tree generated using the same alignment as in (C). In all analyses the amphioxus gene groups strongly with its vertebrate or sea urchin cognates, supporting orthology. Bootstrap values and quartet-puzzling reliability scores are shown at the branch points, and distance units are shown in the upper right hand or lower left hand corners of the trees. Gene names are prefixed by the initials of their respective species names. Species included in the analyses are *Drosophila melanogaster, Nematostella vectensis, Branchiostoma floridae, Ciona intestinalis, Danio rerio, Gallus gallus,* and *Mus musculus*.

**Figure S5.** Phylogenetic analyses of amphioxus *Twist.* (A) Bootstrapped Neighbor-Joining tree of chordate, cnidarian, and *Drosophila Twist* homologs. (B) Maximum Likelihood tree generated using the same alignment as in (A). In both analyses, amphioxus *Twist* groups with vertebrate *Twist* genes at high confidence values. Bootstrap values and quartet-puzzling reliability scores are shown at the branch points, and distance units are shown in the upper right hand or lower left hand corners of the trees. Gene names are prefixed by the initials of their respective species names. Species included in the analyses are *Drosophila melanogaster, Nematostella vectensis, Branchiostoma floridae, Danio rerio,* and *Mus musculus*.

# Supplemental References

1. Thompson JD, Gibson TJ, Plewniak F, Jeanmougin F, Higgins DG (1997) The CLUSTAL_X windows interface: flexible strategies for multiple sequence alignment aided by quality analysis tools. Nucleic Acids Research 25: 4876-4882.

2. Saitou N, Nei M (1987) The Neighbor-Joining method- a new method for reconstructing phylogenetic trees. Molecular Biology and Evolution 4: 406-425.

3. Perriere G, Gouy M (1996) WWW-query: An on-line retrieval system for biological sequence banks. Biochimie 78: 364-369.

4. Schmidt HA, Strimmer K, Vingron M, von Haeseler A (2002) TREE-PUZZLE: maximum likelihood phylogenetic analysis using quartets and parallel computing. Bioinformatics 18: 502-504.
