# Supplementary figures and images for: Insights from Amphioxus into the Evolution of Vertebrate Cartilage
Source: PLoS One. 2007 Aug 29;2(8):e787. doi: 10.1371/journal.pone.0000787 (PMC1950077; doi:10.1371/journal.pone.0000787)

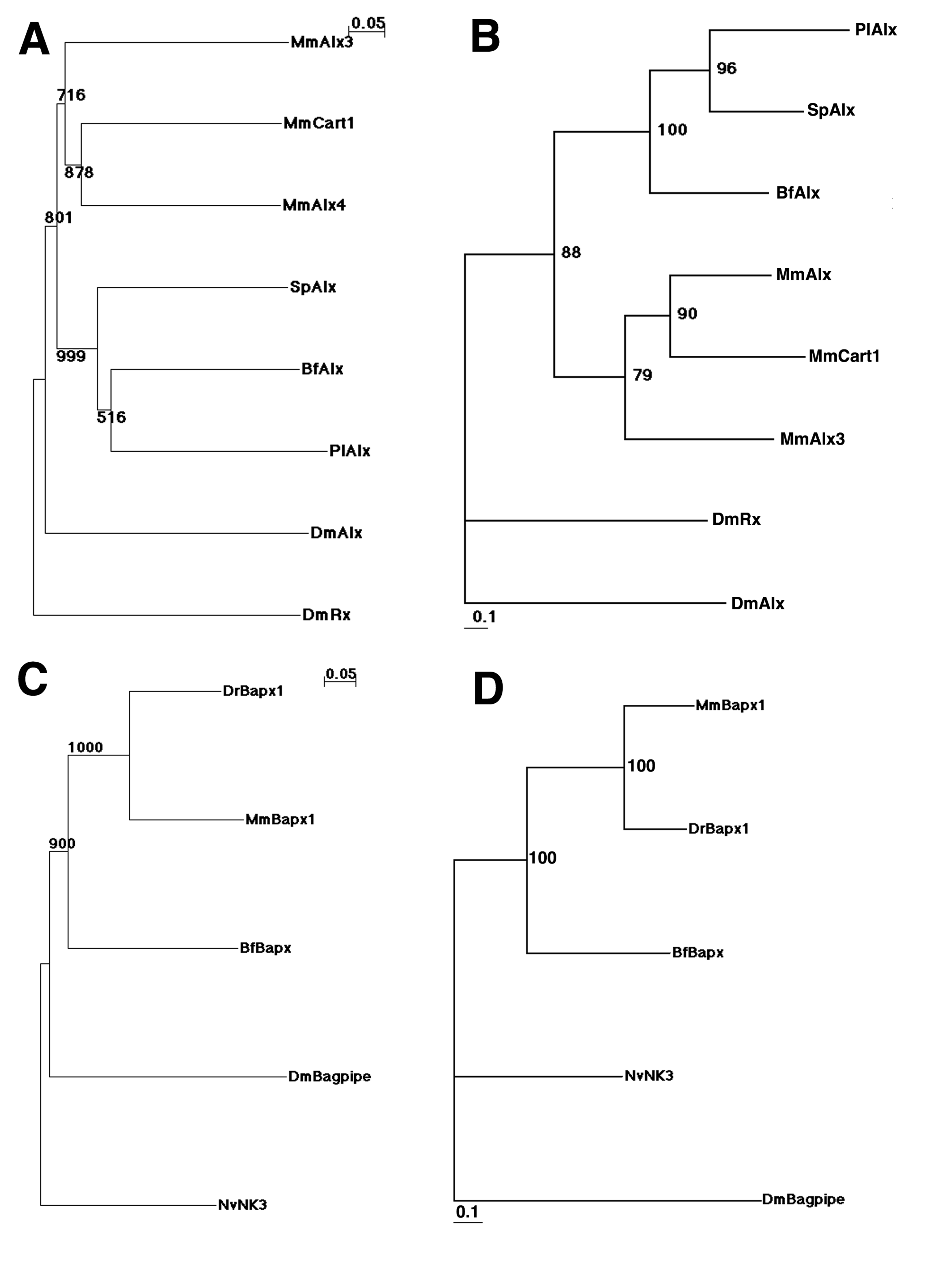

Supplement: Figure S1 — (1.40 MB TIF) [file pone.0000787.s002.tif]

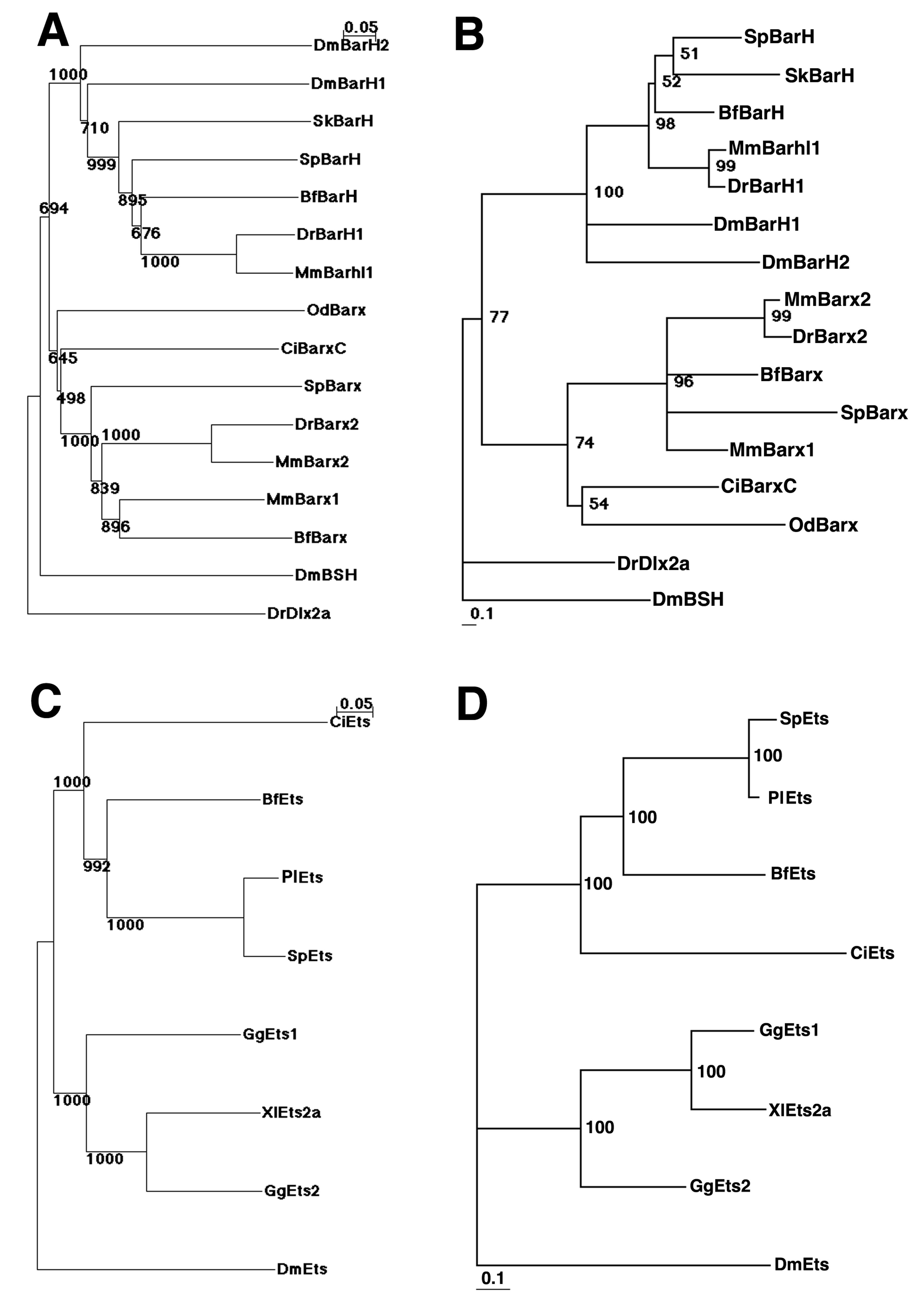

Supplement: Figure S2 — (1.91 MB TIF) [file pone.0000787.s003.tif]

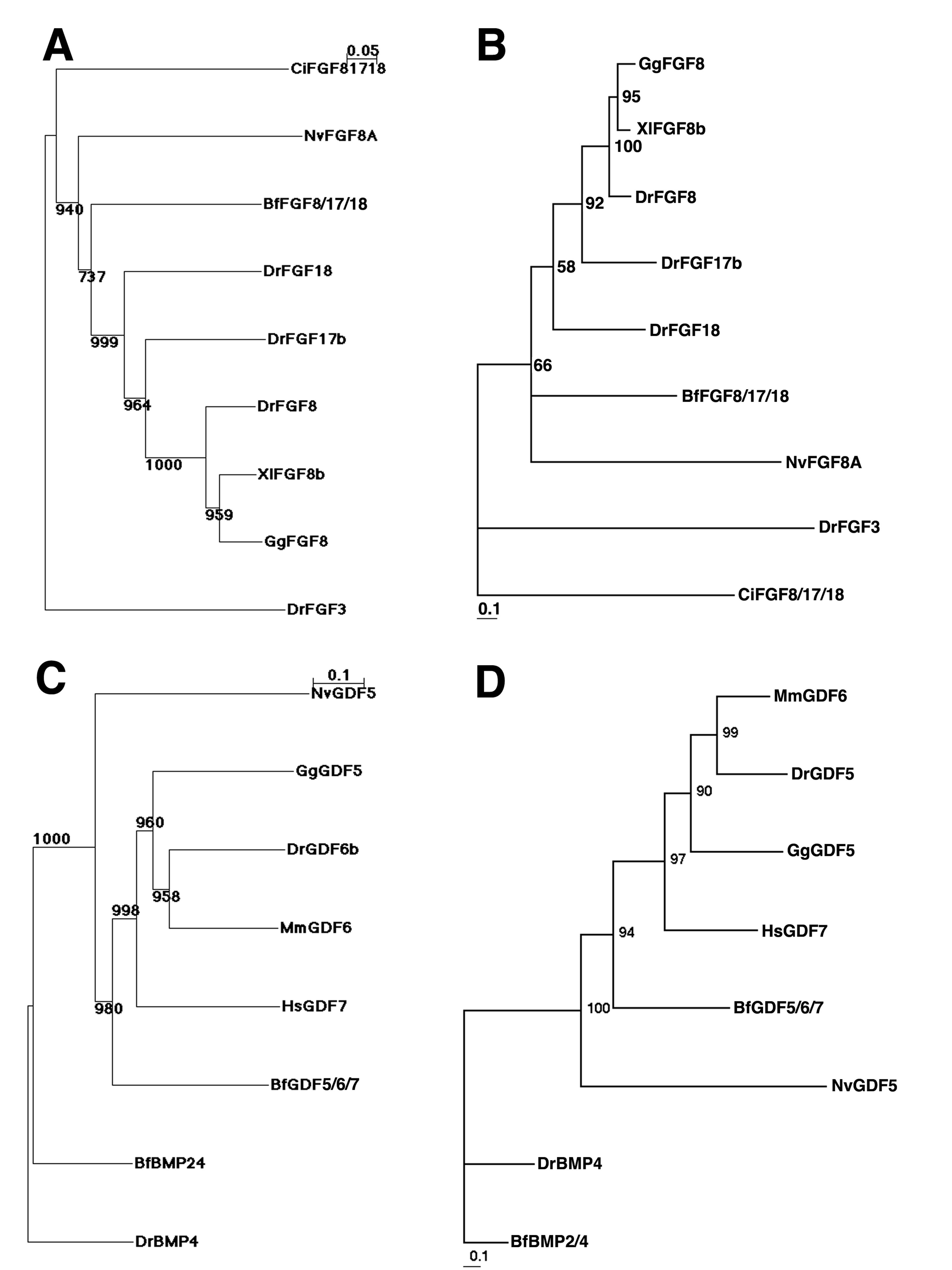

Supplement: Figure S3 — (1.57 MB TIF) [file pone.0000787.s004.tif]

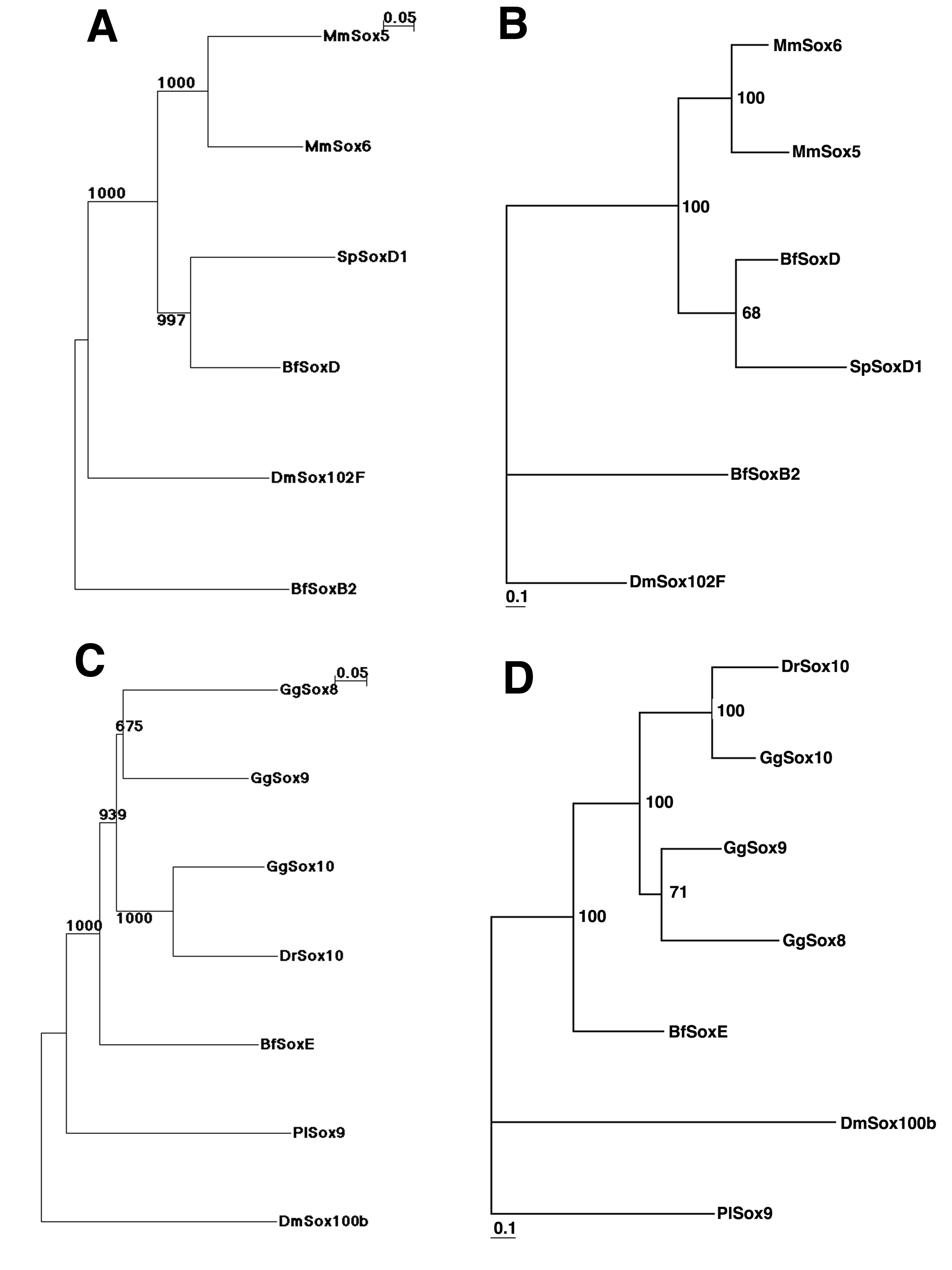

Supplement: Figure S4 — (1.40 MB TIF) [file pone.0000787.s005.tif]

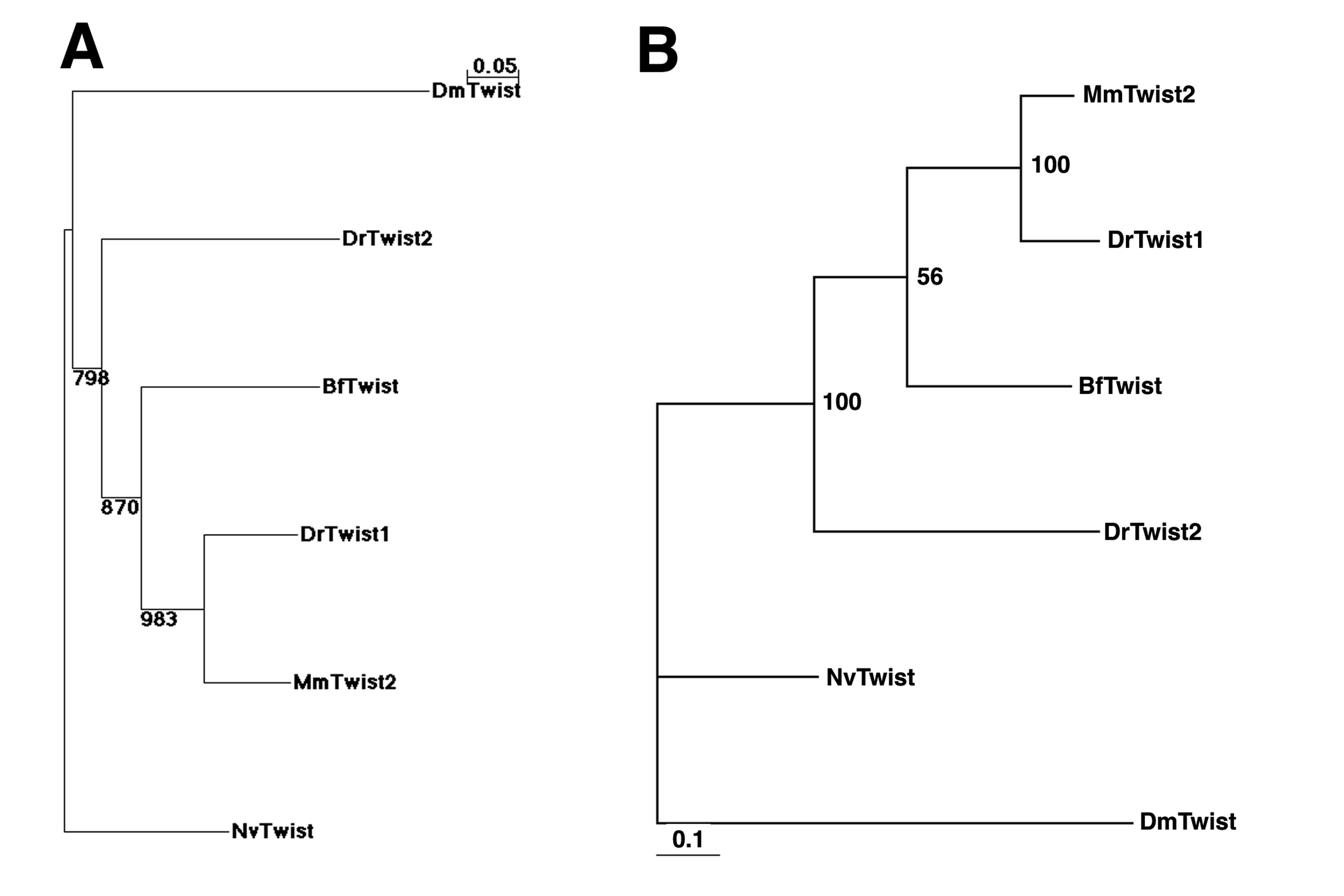

Supplement: Figure S5 — (0.69 MB TIF) [file pone.0000787.s006.tif]
